# Identification of the Female-Produced Sex Pheromone of the Leafminer *Holocacista capensis* Infesting Grapevine in South Africa

**DOI:** 10.1007/s10886-015-0611-9

**Published:** 2015-08-14

**Authors:** Hong-Lei Wang, Henk Geertsema, Erik J. van Nieukerken, Christer Löfstedt

**Affiliations:** Department of Biology, Lund University, SE-223 62 Lund, Sweden; Department of Conservation Ecology and Entomology, Stellenbosch University, Stellenbosch, South Africa; Naturalis Biodiversity Center, Leiden, The Netherlands

**Keywords:** Sex pheromone, Leafminer, Heliozelidae, *Holocacista capensis*, Primitive moth, Vineyard pest, *Vitis vinifera*, (*Z*)-5-tetradecenal, (*Z*)-7-tetradecenal, Lepidoptera

## Abstract

We report the first identification of a sex pheromone in a heliozelid moth, *Holocacista capensis* van Nieukerken & Geertsema. This leafminer recently infested grapevine in South Africa. Compared to solvent extraction of pheromone glands, solid phase microextraction (SPME) proved to be highly effective for collection of the pheromone from calling females. The volatiles collected by SPME were analyzed by gas chromatography with electroantennographic detection (GC/EAD). Three compounds eliciting electrophysiological activity from the male antenna were identified as (*Z*)-5-tetradecenal, (*Z*)-7-tetradecenal, and (*Z*)-9-hexadecenal by coupled gas chromatography–mass spectrometry (GC/MS). GC/MS analysis of dimethyldisulphide (DMDS) derivatives of fatty acyl moieties in pheromone gland extracts confirmed the presence of the corresponding putative pheromone precursors with double bonds in the same position and with *Z* geometry. Field trapping experiments in a South African vineyard confirmed that both (*Z*)-5-tetradecenal and (*Z*)-7-tetradecenal are essential for the attraction of male *H. capensis*, whereas addition of (*Z*)-9-hexadecenal to the blend did not affect the attractiveness. The composition of the pheromone is discussed in relation to the phylogeny of this family of moths.

## Introduction

Leafmining Lepidoptera, such as the globally problematic citrus leafminer *Phyllocnistis citrella* Stainton (Gracillariidae) (Heppner and Dixon 1995), and species of the genus *Phyllonorycter* that attack apple (Maier 2001; Minarro and Jacas 2011) constitute serious economic problems. Leafminers or sometimes gall-makers, particularly those from the poorly-studied Heliozelidae family, can cause damage on host plants belonging to the grape family Vitaceae. This may affect the grape and subsequent production of wine and raisins (Alma 1995; Duso et al. 2011; van Nieukerken et al. 2012; Ueno et al. 1987).

In recent years, a heliozelid leafminer has sometimes occurred in high densities in the table grape growing area near Paarl (Western Cape, South Africa), where the presence of this insect is of concern in relation to export of table grapes. Recently, this leafminer, considered to be a native South African species, has been described as the new species *Holocacista capensis* van Nieukerken & Geertsema. This species probably shifted hosts from native *Rhoicissus* species (Vitaceae) onto the introduced grapevine (van Nieukerken and Geertsema 2015). The moth, which is only about 1.5 to 2 mm long, occurs in several generations throughout the growing season, including the late summer when the grapes are harvested.

To protect grapevines against insect infestation, various pesticides have been used over the past decades with the result that pesticide residues may contaminate the grapes and are eventually carried through into the wine (Andrey and Amstutz 2000; Cabras et al. 1995). The use of pesticides is not recommended, especially in the period around harvest when the leafminer may be highly abundant. In this case, the table grape industry would particularly benefit from other environmentally safe and sustainable ways for control of vineyard pests, and the use of insect pheromones might be a good alternative.

Rigorously identified sex pheromones have so far not been reported in the moth family Heliozelidae, but, in a field screening test in Hungary, male adults of *Antispila treitschkiella* (Fischer von Röslerstamm) were recorded in traps baited with (*Z*)-7-tetradecenal (Z7-14:Ald) (Tóth et al. 1992). This group of moths has not been recognized to contain important pests until recently (Bernardo et al. 2015; van Nieukerken et al. 2012), and consequently, there was no particular incentive to identify their pheromones. Furthermore, collection and isolation of volatiles from these very tiny moths is a challenge, although previous studies in some small leafminers such as the *Phyllonorycter* species have been successful, using the solid phase microextraction technique (SPME) (Borg-Karlson and Mozûraitis 1996).

From a phylogenetic point of view, the structures of the heliozelid sex pheromones are of great interest, because Heliozelidae belongs to Adeloidea, one of the Lepidopteran superfamilies that is not classified to the large clade Ditrysia, but to earlier diverging Lepidoptera. There is a diversity of pheromone structures reported from the few non-ditrysian families where pheromones have been studied, including the Eriocraniidae (Kozlov et al. 1996; Zhu et al. 1995), Nepticulidae (Tóth et al. 1995), Prodoxidae (Löfstedt et al. 2004), and Tischeriidae (Molnár et al. 2012). Here, we report identification of the first heliozelid sex pheromone, that of the grape leafminer, *H. capensis*.

## Methods and Materials

### Insects

Larvae of the summer generation of *H. capensis* were collected in table grape plantations of the cultivars “Regal” and “Red Globe”, North-West of Paarl, (33°43′S, 18°57′E), Western Cape, South Africa, on 25 January 2013 (disclaimer: the sampled farm was not particularly more affected than any nearby estate, whether table grape or wine farm). Leafmines were kept with pieces of the leaf in small plastic containers. After the larvae prepared a cocoon and left the mine, 50 individual cocoons were each placed in a small glass tube (10 x 1 cm i.d.) and sent to Lund where they were stored at 23 °C, 60 % R.H. and 18:6 h L:D cycle. Moths (21 females and 11 males) emerged between 9 and 15 February. Newly emerged moths were separated in individual vials (preventing mating) and sexed based on the difference in relative length of antenna. The adults were kept individually until used in experiments.

### Chemicals

Reference compounds of different origin and purity were available for the identification work from our laboratory collection of pheromone compounds. For chemical identification and electrophysiological experiments, (*Z*)-5-tetradecenal (Z5-14:Ald), (*E*)-5-tetradecenal (E5-14:Ald), (*Z*)-7-tetradecenal (Z7-14:Ald), and (*E*)-7-tetradecenal (E7-14:Ald) were prepared by oxidation of corresponding alcohols with pyridinium chlorochromate as described by Corey and Suggs (1975). The corresponding fatty acids and their methyl esters also were prepared from the respective alcohols as described in Wang et al. (2010). Z5-14:Ald (chemical purity 99.6 %), Z7-14:Ald (chemical purity 98.2 %), and (*Z*)-9-hexadecenal (Z9-16:Ald, chemical purity 99.0 %) used for field experiments were purchased from Pherobank (Wageningen, The Netherlands).

### Sex Pheromone Collection

SPME samples were collected from 1 to 7 d-old virgin female adults. Thus, 7–9 virgin females were put in a 5 ml gas tight syringe (Hamilton, Switzerland) into which a fiber coated with PMDS (7 μm film thickness, Supelco, USA) was inserted. Headspace volatiles were collected from the same batch of females for the first 6 hr (scotophase), the next 12 hr (photophase), the next 24 hr, and a final 48 hr. The SPME samples were subjected immediately to GC/MS or GC/EAD analysis.

In parallel to the SPME collection, batches of female abdominal tips, where the pheromone gland is assumed to be located, were dissected from virgin females up to 2 d-old during three different periods, *i.e*., 2–4 hr into scotophase, 1 hr before scotophase, and 1 hr before photophase. Either a single abdominal tip or up to nine combined tips were extracted with 10 μl of redistilled hexane and either analyzed by GC/MS or used for the GC/EAD assay.

The fatty acyl pheromone precursors in the abdominal tip residuals were subsequently extracted in 10 μl chloroform/methanol (2:1 v:v) at room temperature for 24 hr, and then analyzed by GC/MS as corresponding methyl esters after basic methanolysis as described in Bjostad and Roelofs (1984).

### Electrophysiology

An Agilent 7890 gas chromatograph equipped with an HP-INNOWax column (30 m × 0.25 mm i.d., and 0.25 μm film thickness; J&W Scientific, USA) and a flame ionization detector (FID), as well as an electroantennographic detector (EAD) was used to identify physiologically active compounds in female pheromone extracts. The GC inlet was set at 230 °C, and a split of the carrier gas (hydrogen) at the end of the column allowed a 1:1 division of the GC effluent between the FID and EAD. After cutting off the tips, the antennae together with the head of 1-2-d-old male adults were mounted to a PRG-2 EAG (10x gain) probe (Syntech, Kirchzarten, Germany) using conductive gel (Blågel, Cefar, Malmö, Sweden). Charcoal-filtered and humidified air passed over the antennal preparation through a glass tube outlet connecting to the GC transfer line, which was heated at 255 °C. The temperature of the GC oven was programmed from 80 °C for 1 min, then at 10 °C/min to 210 °C, held for 10 min and then to 230 °C at 10 °C/min and held for another 10 min. For SPME samples, the fiber was inserted into the GC inlet and held for 5 min. Data were analyzed with the software GC-EAD Pro Version 4.1 (Syntech, Kirchzarten, Germany).

### Gas Chromatography/Mass Spectrometry

An Agilent 5975 mass-selective detector coupled to an Agilent 6890 gas chromatograph was used to analyze the pheromone gland extracts, SPME samples, and the fatty acyl pheromone biosynthetic precursors. An HP-INNOWax column (as above), and a non-polar column HP-5MS (30 m × 0.25 mm i.d., and 0.25 μm film thickness; J&W Scientific, USA) were used. Carrier gas was helium at a constant flow of 0.8 ml/min corresponding to linear velocity of 33 cm/s. For analysis of most samples, the oven temperature was programmed as in the GC/EAD analysis. However, to separate better the Z5-14:Ald and E5-14:Ald isomers, a slower oven temperature program was set with the HP-INNOWax column as 60 °C for 1 min, then at 4 °C/min to 200 °C, and then to 230 °C at 10 °C/min and held for 15 min. For the analysis of DMDS-adducts of methanolized fatty acyl precursors, the HP-5MS column was used, and the oven temperature was programmed from 80 °C for 2 min, then at 15 °C/min to 140 °C, and then to 260 °C at 5 °C/min and held for 20 min.

Compounds were identified based on comparison of their retention times and mass spectra with those of synthetic references on both polar and non-polar columns. Double bond positions in the fatty acyl biosynthetic precursors were localized by GC/MS analysis of the dimethyldisulphide adducts of corresponding methyl esters, prepared according to Dunkelblum et al. (1985).

### Field Experiments

Field trapping experiments were carried out from 22^th^ March to 7^th^ May 2013 in the Opdenhorst farm (33°43′S 18°58′E) in the Paarl district (disclaimer: this farm was not particularly more affected than any nearby estate, whether table grape or wine farm.). Synthetic blends prepared in various dose and composition in hexane were loaded on rubber septa (Catalogue no. 224100–020, Wheaton Science Products, Millville, NJ, USA) as lures. Butylated hydroxytoluene (BHT, 0.02 %) was added to the hexane as antioxidant to extend the field life of the aldehydes.

Treatments including a negative control with hexane alone were evaluated in two experiments. The first experiment was designed to determine the optimal dosage of a ternary blend containing all the three identified GC/EAD active components, Z5-14:Ald, Z7-14:Ald, and Z9-16:Ald (baits A-D). The blend ratio was set as found in the SPME collection, *i.e*., 33 : 100 : 12, respectively. The second experiment (baits F-H) was designed to include treatments with one component subtracted at the second highest dosage used in the first group. For each treatment there were five replicates. A positive control (bait C) was used in both groups to allow comparison between groups. The actual blend ratios in all the treatments were confirmed by GC/FID analysis on an HP-INNOWax column before the field test.

Delta-traps with pheromone lures and sticky inserts (Csalomon, Budapest, Hungary) were suspended just below the vine canopy and spaced 15 m apart in two rows also 15 m apart in five sites, each at least 100 m apart, with the pheromone treatments in random sequences. Traps were inspected once a week and redistributed within each replicate so as to minimize the potential position effect. The trapped moths were identified by their morphological characteristics.

### Statistical Analyses

Differences in trap catch between treatments were compared using one-way ANOVA analysis on log (x + 1) transformed data, followed by a Least Significant Difference (LSD) test (*P* < 0.01). All analyses were performed using SPSS ver.16.

## Results

### Pheromone Identification

**Fig. 1:**
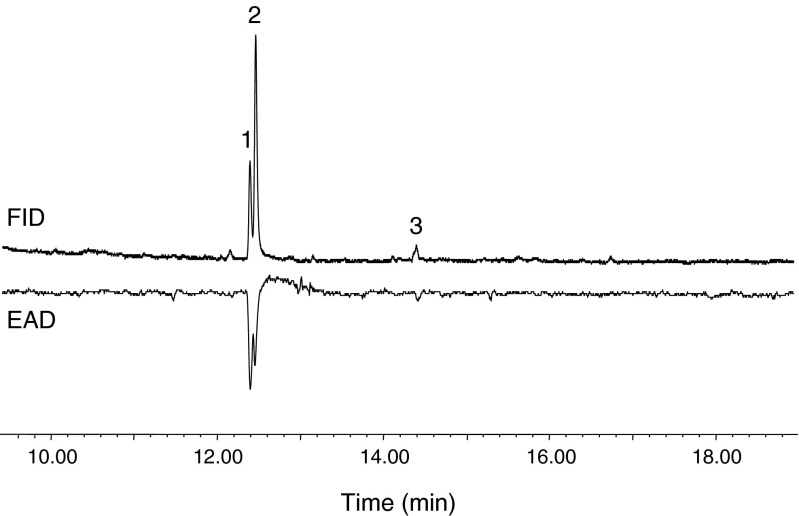
Coupled gas chromatographic-electroantennographic analysis of volatiles from female *Holocacista capensis* collected by SPME. The *upper trace* is the chromatogram monitored with flame ionization detector (FID), and the *lower trace* is the antennal response from conspecific male antennae. The SPME sample was collected for 24 hr from 8 virgin females at age of 4–5 d-old

Analysis of the hexane extracts of female abdominal tips from *H. capensis* by GC/EAD did not reveal any EAD-active peaks. As an alternative approach, SPME proved to be highly effective for the collection of the pheromone from live female adults, even when sampling from a small number of individuals. GC/EAD analyses of the SPME samples showed clearly that the male antennae responded to three components in the collections from conspecific females (Fig. 1). Compounds 1 and 2 eluted close to each other in relatively large amounts and elicited large antennal responses, whereas compound 3 was present in much smaller amounts and evoked a smaller antennal response. These active components were considered as potential pheromone components and identified by GC/MS analysis.

**Fig. 2:**
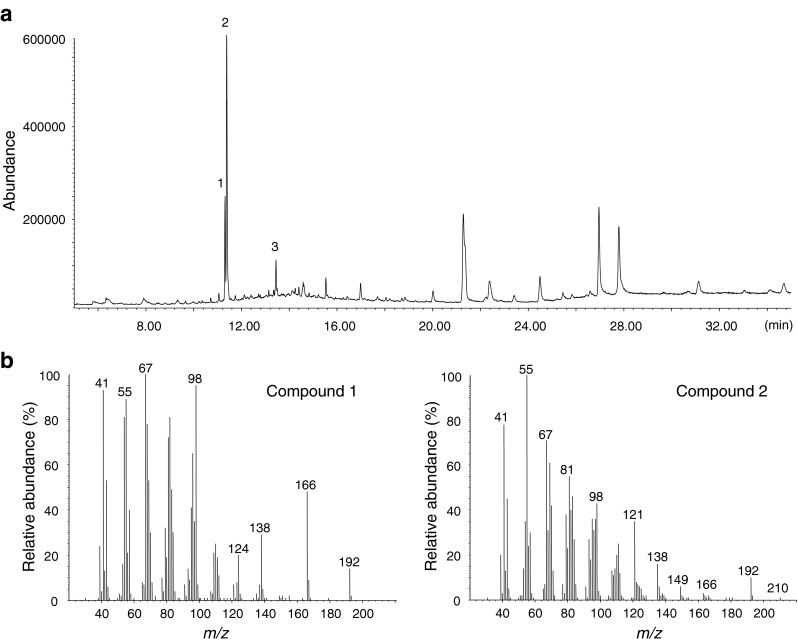
GC/MS analysis of SPME collection from female *Holocacista capensis*. (**a**) total ion current (TIC) chromatogram. The SPME sample was collected for 48 hr from 9 virgin females at age of 2–4 d-old. (**b**) mass spectra of GC/EAD-active compounds 1 and 2

The mass spectrum of the most abundant compound 2 showed a tentative molecular ion at *m*/*z* 210, a fragment of [M-18]^+^ at *m*/*z* 192, and a relatively less abundant fragment of [M-44]^+^ at *m*/*z* 166. These diagnostic ions suggested a possible structure of a mono-unsaturated C_14_ aliphatic aldehyde (Fig. 2). Compared to component 2, the mass spectrum of component 1 exhibited the same fragments, except for the missing molecular ion at *m*/*z* 210. However, the ion ratios were very different between the two spectra, implying that the two components might have similar structures but different double bond locations. The ions at *m*/*z* 166 and *m*/*z* 98 were particularly prominent in the spectrum of compound 1 (Fig. 2), and the latter fragment was previously reported as a diagnostic ion in the 5,7- and 7,9- conjugated hexadecadienyl compounds (Nishida et al. 2003).

**Fig. 3:**
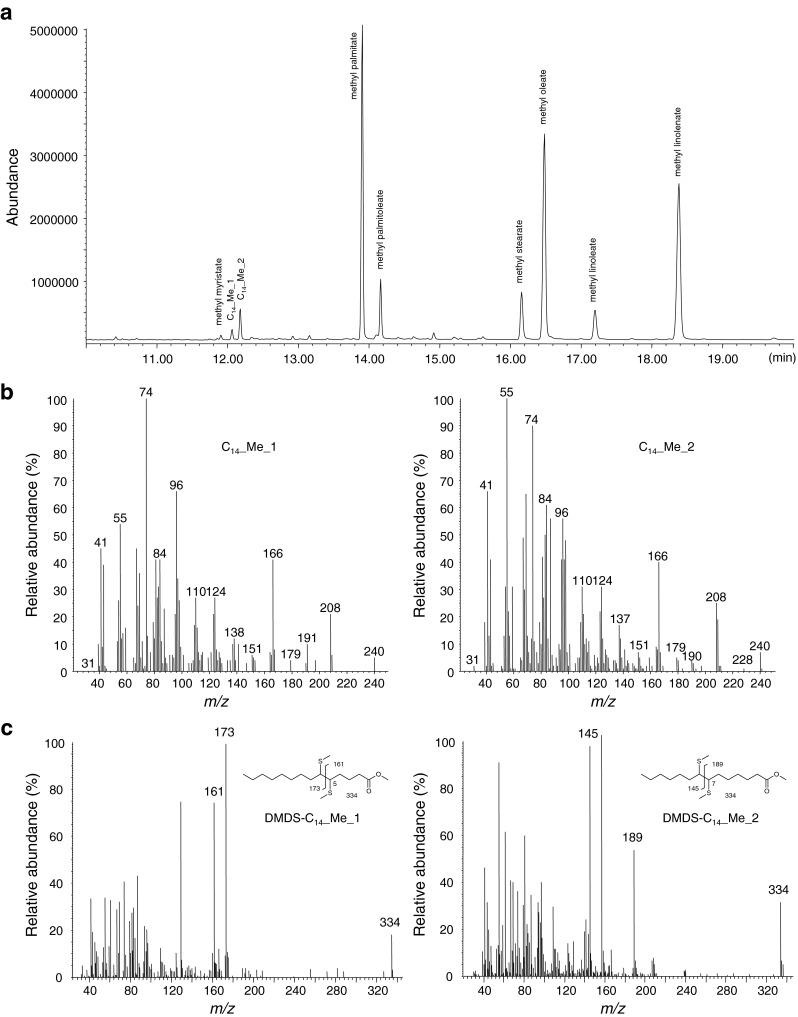
GC/MS analysis of pheromone fatty acyl biosynthetic precursors from pheromone glands of *Holocacista capensis* in form of their methyl esters. (**a**) total ion current (TIC) chromatogram of gland fatty acyl methyl esters; (**b**) mass spectra of the two C_14_ mono-unsaturated methyl esters; (**c**) mass spectra of DMDS-adducts showing a double bond at 5- and 7- position in the C_14__Me_1 and C_14__Me_2, respectively

As it was not possible to perform DMDS derivatization directly on the SPME collections, we analyzed potential fatty acyl biosynthetic precursors extracted from the abdominal tips and subjected to basic methanolysis. GC/MS analyses of the methyl esters showed in addition to the ubiquitous fatty acids myristic, palmitic, palmitoleic, stearic, oleic, linoleic, and linolenic acids, two mono-unsaturated C_14_ acids (Fig. 3a, b). GC/MS analysis of the DMDS adducts of the methyl esters revealed two C_14_ methyl esters exhibiting double bonds in different positions. One compound had a double bond between carbon atoms 5 and 6 in the acyl skeleton, as indicated by a pair of diagnostic ions at *m*/*z* 161/173, and a molecular ion at *m*/*z* 334. In the other compound, the double bond was between carbon atoms 7 and 8, as indicated by the ion pair at *m*/*z* 189/145, and a molecular ion at *m*/*z* 334 (Fig. 3c). These two esters were identified as methyl (*Z*)-5-tetradecenoate (Z5-14:Me) and methyl (*Z*)-7-tetradecenoate (Z7-14:Me) by comparing their mass spectra and retention times with those of the reference compounds. These results suggested that the mono-unsaturated C_14_ aldehydes in the SPME samples should have a double bond at 5- and 7- positions, respectively. These structures were verified by comparing the mass spectra and GC retention times of the insect-derived compounds with corresponding data for synthetic Z5-14:Ald and Z7-14:Ald, and their geometric isomers E5-14:Ald and E7-14:Ald. The position and configuration of the double bond was confirmed as Z5 in compound 1 and Z7 in compound 2.

The mass spectrum of component 3 showed a putative molecular ion at *m*/*z* 238, a fragment of [M-18]^+^ at *m*/*z* 220, and a fragment of [M-44]^+^ at *m*/*z* 194, indicating a mono-unsaturated C_16_ aldehyde. Correspondingly, a C_16_ monounsaturated acid was found from the DMDS adducts of the fatty acid methyl esters. The related mass spectrum exhibited a pair of diagnostic ions at *m*/*z* 145/217 and a molecular ion at *m*/*z* 362, thus indicating a double bond between carbon atoms 9 and 10. Consequently, compound 3 in the SPME samples was identified as Z9-16:Ald by comparing the mass spectrum and retention time of the natural product with corresponding data of synthetic (*E*)-9-hexadecenal and Z9-16:Ald on two columns.

The amounts of the three EAD-active components, 1, 2, and 3, obtained in SPME collections were estimated as 1.1, 2.9, and 0.4 ng, respectively, based on the comparison of the peak area with the reference compounds of known concentration. The synthetic Z5-14:Ald, Z7-14:Ald and Z9-16:Ald elicited strong responses from the antennae of male *H. capensis* in GC/EAD analyses.

### Field Tests

**Fig. 4:**
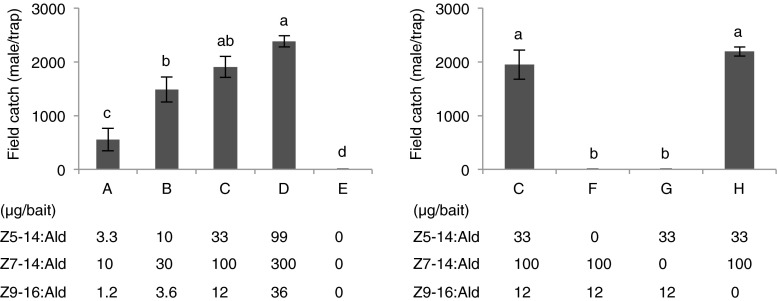
Trap catches of male *Holocacista capensis* by different dosages and blends of candidate pheromone components in a table grape vineyard in Stellenbosch, South Africa. *Bars* with the same letters indicate means that are not significantly different at *P* < 0.01, one-way-ANOVA on log (x + 1)-transformed data followed by a LSD test; 5 replicates for each treatment

To test the behavioral activity of the pheromone candidates, field-trapping experiments were carried out in a plantation with table grapes in South Africa. In the first experiment, the blend of Z5-14:Ald, Z7-14:Ald, and Z9-16:Ald at 33 + 100 + 12 μg/bait in the ratio found in the SPME samples, was more attractive to *H. capensis* males than the lower doses, and equally good as the three times higher dose. In the second experiment, the binary blend of Z5-14:Ald and Z7-14:Ald in a ratio of 1:3 was as attractive as the ternary bland to male *H. capensis*, whereas subtraction of either Z5-14:Ald or Z7-14:Ald from the ternary blend decreased the trap catch, indicating that both tetradecenals are essential to the attractiveness (Fig. 4). This two-component sex pheromone was highly effective in attracting *H. capensis*. In the case of the optimized blend and dose (treatment H in Fig. 4), more than 2000 males/trap were caught in the first week of the field experiment.

## Discussion

We identified the sex pheromone of *H. capensis* as a binary blend of Z5-14:Ald and Z7-14:Ald. The combination of SPME, GC/EAD, and GC/MS proved to be effective for the collection and identification of the sex pheromones from small heliozelid moths.

According to the Pherobase (www.pherobase.com including original references), Z7-14:Ald was identified previously as a sex pheromone component in some *Prays* species belonging to the relatively primitive ditrysian moth family Praydidae (Yponomeutoidea). It also was attractive to a few noctuid species, such as *Spaelotis clandestina* (Harris) and *Simyra henrici* (Grote) (Steck et al. 1982a, b). As previously mentioned, Z7-14:Ald was recorded as an attractant for a heliozelid species *Antispila treitschkiella* in a field screening experiment using synthetic lepidopteran pheromone compounds (Tóth et al. 1992). Compared to Z7-14:Ald, Z5-14:Ald has not been reported as a moth pheromone component, although it was reported as sex attractant to the noctuid moth *Agrotis volubilis* Harvey in a field survey, in which a set of C_12_, C_14_ and C_16_ straight olefinic aldehydes and aldehyde-acetate mixtures were used as candidate lures (Underhill et al. 1977).

In this study, solvent extracts of the pheromone glands did not contain the pheromone components but SPME collections did. As a strategy to avoid the potential cell toxicity caused by the accumulation of the reactive aldehydes, the females might release the compounds immediately after their biosynthesis. It also is possible that the time for dissecting the female abdominal tips in this study was not optimal, due to the lack of knowledge about the exact pheromone-releasing period in *H. capensis*. The relative amounts of the three components observed in SPME collections were not necessarily the actual ratios present in the pheromone gland or even those released from the gland, due to the probable different affinities of the compounds and the fiber. In the present study, the C_14_ and C_16_ monoene aldehydes would be expected to differ significantly in their affinities for the fiber, whereas discrimination between the two C_14_ aldehyde isomers, Z5-14:Ald and Z7-14:Ald, would be much less because of their close physicochemical properties.

The two methyl esters, Z7-14:Me and Z5-14:Me found in the pheromone gland extracts after methanolysis of fatty acyl pheromone precursors may be derived by limited chain-shortening (β-oxidation) of palmitoleic acid ((*Z*)-9-hexadecenoic acid) and oleic acid ((*Z*)-9-octadecenoic acid), respectively. This proposed pathway is in agreement with what has been confirmed for the predominant type I pheromones, *i.e*., long-chain fatty alcohols and corresponding aldehydes and acetates, in higher lepidopteran species. It remains to be investigated whether the desaturase involved in this pathway belongs to the ubiquitous metabolic ∆9 desaturases, which produce palmitoleic and oleic acids, or the Lepidoptera-specific ∆9 desaturase subfamily that is exclusively used for pheromone production (Liénard et al. 2008). An alternative, less parsimonious pathway, requiring two unusual desaturases directly acting at the 7- and 5- positions of myristate, cannot be positively excluded without performing the appropriate experiments.

**Fig. 5:**
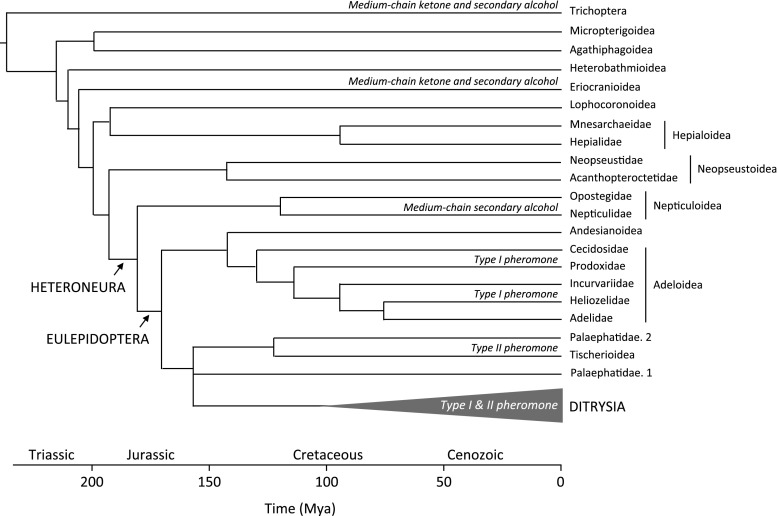
Differentiation of lepidopteran sex pheromones based on chemical structures. Phylogeny of early lepidopteran superfamilies with estimated time of divergence is retrieved from Regier et al. (2013, 2015) and Wahlberg et al. (2013). Type I pheromone refers to the long-chain fatty alcohols, aldehydes, and acetates. Type II pheromone refers to the C_17_-C_23_ polyenes and corresponding epoxides. For the sake of simplicity, the new monotypic families Aenigmatineidae and Tridentaformidae are left out. The Palaephatidae are paraphyletic with regard to Tischerioidea (Regier et al. 2015)

Heliozelid moths belong to the superfamily Adeloidea (formerly Incurvarioidea), which are among the lepidopteran lineages that diverged before the origin of the Ditrysia to which more than 98 % of Lepidoptera belong (Fig. 5) (van Nieukerken et al. 2011; Regier et al. 2015). The currently known female-produced sex pheromones found in the non-ditrysian moths fall into three distinct types according to their chemical characteristics. One type, found in the adeloid species, *e.g*., *Lampronia capitella* (Clerck), family Prodoxidae (Löfstedt et al. 2004), and *H. capensis*, family Heliozelidae (current study), comprise unsaturated fatty alcohols, aldehydes, and acetates, which are typical so called Type I moth pheromones (Ando et al. 2004). The long-chain polyenes of the tischeriid *Tischeria ekebladella* (Bjerkander) (Molnár et al. 2012) are structurally similar to the Type II pheromones identified in the Ditrysia (Millar 2000). The third type consists of chiral, medium-chain secondary alcohols and their corresponding ketones. These pheromones are found in some nepticulid species, *e.g*., *Stigmella malella* (Stainton) and *Trifurcula melanoptera* van Nieukerken & Puplesis (Tóth et al. 1995), and in the even earlier diverging lineage Eriocranioidea (Zhu et al. 1995) as well as in the caddisflies from the sister order Trichoptera (Löfstedt and Kozlov 1997). According to recent phylogenetic studies (Regier et al. 2013, 2015; Wahlberg et al. 2013), the Tischeriidae form together with the Palaephatidae the sister group of the Ditrysia, whereas the Adeloidea and Andesianoidea are sister groups to the above combination (Fig. 5). The Nepticuloidea branch off just before the Adeloidea, and have the primitive pheromone type, suggesting that the common sex pheromones of the extant ditrysian moths might have evolved in the stemgroup of Adeloidea/Andesianoidea and remaining Lepidoptera. Identification of a sex pheromone from a species in Andesianoidea, the sister group to the Adeloidea, in such a case would be interesting and possibly corroborate this suggestion.

The highly attractive pheromone lure for *H. capensis* reported in this study will be useful to perform monitoring and large-scale control of this secondary vineyard pest. Furthermore, identification of the pheromone of this primitive moth species provides further insight into the early evolutionary history of lepidopteran pheromones.
